# Decoding Hidden Pathways: A Comprehensive Exploration of Anomalous Coronary Arteries

**DOI:** 10.7759/cureus.86852

**Published:** 2025-06-27

**Authors:** Aineesh Vallurupalli, Rajesh Thachathodiyl

**Affiliations:** 1 Department Of Cardiology, Amrita Institute of Medical Sciences and Research Centre, Kochi, IND; 2 Department of Cardiology, Amrita Institute of Medical Sciences and Research Centre, Kochi, IND

**Keywords:** alcapa, computed tomography angiography (cta), coronary artery anomalies, interarterial left main, sudden cardiac death

## Abstract

Introduction

Coronary artery anomalies (CAA) are congenital anomalies affecting the origin, course, structure, or number of coronary arteries; certain high-risk configurations, particularly those involving interarterial or intramural segments, can predispose individuals to ischemia, arrhythmias, or sudden cardiac death (SCD). Populations at particular risk include young athletes and individuals with anomalies exhibiting interarterial or intramural courses. This study aimed to characterize the prevalence, imaging features, management strategies, and outcomes of CAAs, including rare variants such as anomalous left coronary artery from the pulmonary artery (ALCAPA), and interarterial left main in a single tertiary care center cohort.

Methods

We retrospectively analyzed 8,000 patients who underwent coronary angiography between January 2012 and December 2021 and identified 133 patients with CAAs. Diagnostic modalities included invasive coronary angiography (CAG) for typical ischemic or acute presentations and coronary CT angiography (CTA) for atypical or lower-risk cases; MRI was unavailable. Demographic data, clinical features, anomaly subtypes, and management strategies [medical therapy, percutaneous coronary intervention (PCI), or surgery] were documented. Outcomes at a median 36-month follow-up included symptom resolution and adverse events clearly defined as myocardial infarction (Fourth Universal Definition), revascularization [PCI/coronary artery bypass grafting (CABG) for stenosis >60%], and cardiac death.

Results

A total of 133 patients (mean age: 52.3 ± 10.5 years; 62% male) were included. Chest pain (60%) was the most common symptom, with 10% asymptomatic. The most frequent anomalies were classified as benign variants, including anomalous right coronary artery (RCA) from the left sinus (37.6%) and left circumflex artery (LCx) from the right sinus (30.1%). Malignant variants included five cases of ALCAPA (3.8%), two interarterial left main origins (1.5%), and two single coronary artery cases (1.5%). One patient with an interarterial left main died of SCD during follow-up. Other less common forms (e.g., high takeoff, fistulas, significant myocardial bridging) were grouped under “Other” (7.5%). Significant concomitant coronary artery disease (CAD) (>50% stenosis) was present in 17.5%. Management strategies included medical therapy (60%), PCI (22.5%), and surgical correction (17.5%). Symptom resolution occurred in 70% of medically treated, 80% of PCI-treated, and 100% of surgically treated patients. Adverse events (overall 10%) - defined as myocardial infarction, revascularization, or cardiac death - occurred in 5% of the medical group, 8.9% of PCI, and 4.3% of surgical cases. Outcome data for rare variants were limited due to low sample size, but suggest higher risk with malignant anatomies.

Conclusions

Anomalous coronary arteries show wide clinical variability; high-risk types like interarterial left main and ALCAPA warrant careful follow-up. While most patients achieve good outcomes, SCD in one case in our cohort highlights the need for timely intervention. Current challenges include limited awareness, lack of standardized screening, inconsistent risk assessment, and absence of personalized treatment protocols. Advancements in imaging, genetics, and artificial intelligence (AI)-driven risk models may enhance early detection and tailored management of CAAs.

## Introduction

Coronary artery anomalies (CAAs) are a diverse group of congenital abnormalities involving the origin, course, or termination of the coronary arteries, with an estimated incidence of 0.2% to 1.3% in adults undergoing angiography [1,2]. Although many CAAs are benign and asymptomatic, and often detected incidentally during coronary angiography (CAG) or autopsy, they can carry significant clinical implications, particularly in younger populations [1,3,4]. For example, separate origins of the left anterior descending artery (LAD) and left circumflex artery (LCx) or an LCx arising from the right sinus are typically considered benign [1,2], while more complex anomalies may contribute to ischemia, arrhythmias, or sudden cardiac death (SCD) [3-6].

High-risk or “malignant” anomalies, such as anomalous aortic origin of a coronary artery (AAOCA) with interarterial course and anomalous left coronary artery from the pulmonary artery (ALCAPA), are associated with major adverse events, including myocardial infarction, heart failure, and SCD, especially in young athletes [4-6]. Autopsy data show that 85% of SCD cases from coronary anomalies involve interarterial courses of the left main from the right sinus [4]. In contrast, while an anomalous right coronary artery (RCA) from the left sinus is more frequently encountered, its clinical impact tends to be less severe due to a milder ischemic burden or delayed onset [5-8].

Existing studies, such as the seminal work by Yamanaka and Hobbs [1] and more recent CT-based series [2,9], have focused primarily on prevalence and anatomical description, often without detailing real-world outcomes, particularly in rarer subtypes. Additionally, broader CAA definitions in some reports (e.g., including myocardial bridging) have led to variability in prevalence estimates (up to 5-6%) [10,11], though our study excludes such borderline variants to maintain clinical relevance. While advancements in coronary CT angiography (CCTA) have enhanced anatomical resolution and detection rates [8,9], prior studies have not consistently linked imaging findings with clinical decision-making, treatment outcomes, or long-term symptom evolution.

In light of this, we conducted a retrospective, real-world analysis of CAAs diagnosed over 10 years at a tertiary care center to address the following objectives: (1) estimate the prevalence of CAAs in a contemporary adult cohort undergoing coronary imaging; (2) classify the anatomical variants and define their clinical significance based on current risk stratification frameworks; and (3) evaluate outcomes, including symptom resolution, adverse events, and therapeutic strategies across different management pathways.

This study specifically contributes to the literature by reporting detailed outcome data in rare variants, clarifying the clinical impact of high-risk subtypes, and highlighting how imaging modality choice influenced diagnosis and management. In doing so, it expands on previous registry-based studies [1,2,12] by providing a focused, outcome-driven perspective from a South Asian center, with appropriate management of potentially high-risk variants [13,14].

## Materials and methods

Study design and population

We performed a retrospective observational study in the cardiology department of Amrita Institute of Medical Sciences, Kochi, Kerala, India. This center was selected for the study due to its large patient throughput and broad case mix, creating an optimal environment to assess the various clinical manifestations of coronary artery anomalies. Due to the retrospective design of this analysis, the requirement for informed patient consent was waived. We conducted a systematic review of all invasive coronary angiograms (n=8,000) and CCTA studies performed between January 1, 2014, and January 1, 2024, to ensure accurate estimation of the prevalence and comprehensive identification of CAAs. This approach enabled a thorough and unbiased assessment, independent of previously reported findings. Patients of all ages undergoing angiographic evaluation were included. No specific exclusion criteria were applied, aside from the exclusion of poor-quality imaging data that could obscure coronary origin assessment.

Definitions

For this study, a CAA was defined as any congenital deviation from the standard coronary arterial anatomy, which comprises two coronary ostia: the left main artery (bifurcating into the LAD and LCx) arising from the left sinus of Valsalva, and the RCA originating from the right sinus and coursing along the right atrioventricular groove. We included anomalies of origin and course (e.g., origin from the opposite sinus, multiple ostia, single coronary artery, high take-off, ectopic origin from the pulmonary artery) as well as anomalies of termination (e.g., coronary artery fistulae). All detected anomalies were classified into major subtypes (e.g., absent left main, anomalous LCx, anomalous RCA, single coronary artery, etc.) using the Angelini classification system, supplemented by the criteria described by Yamanaka and Hobbs, to enable standardized categorization and facilitate comparison with existing literature. Poor-quality imaging was defined as images insufficient for clear identification or confident categorization of coronary artery origins and anomalies due to artifacts or incomplete visualization. Image quality was assessed independently by two experienced cardiologists/radiologists, with consensus decision-making resolving discrepancies.

Methodology

Each angiographic and CCTA image was independently reviewed by two cardiologists/radiologists. Any discrepancies were adjudicated by consensus involving a third senior specialist. For each patient with a confirmed anomaly, we reviewed medical records and imaging (invasive angiograms or CCTA). We noted demographic data (age, sex), indication for angiography (chest pain, ischemia evaluation, or other reasons), and whether the anomaly was discovered incidentally or linked to the presenting complaint. Specific anomaly types were documented in detail (including interarterial or intramural courses). Risk categorization into benign and malignant anomalies was performed based on anatomical and clinical criteria from widely accepted classifications and guidelines (Angelini [4], Brothers et al. [6], and Cheezum et al. [12]), where interarterial or intramural courses were considered high-risk due to their established association with adverse clinical outcomes. We also recorded any additional cardiac findings, such as coexisting atherosclerotic coronary disease, valvular abnormalities, or structural defects. Importantly, we documented clinical management (observation, PCI, or surgery) and outcomes (e.g., postoperative status, myocardial infarction, survival). Clinical follow-up information was obtained from comprehensive electronic medical records, with all outcomes such as myocardial infarction, revascularization procedures, cardiac death, and symptom resolution confirmed through detailed chart reviews. Follow-up extended until the end of the study period (January 1, 2024).

Statistical analysis

Due to the heterogeneity of anomalies and limited subgroup sizes, inferential statistical analyses were not performed; descriptive statistical analysis was deemed most appropriate to characterize prevalence, clinical presentations, and outcomes. We calculated the prevalence of CAAs as a proportion of all coronary angiograms performed. We tabulated the frequency of each anomaly subtype, and categorical variables (e.g., presence of symptoms, proportion receiving surgery) were summarized as percentages. Where appropriate, we compared our results with previously published data on CAA incidence, classification, and outcomes.

## Results

Study population

Over the 10-year study period, 8,000 patients underwent coronary angiography at our institution. The predominant indication was evaluation of chest pain or suspected myocardial ischemia (e.g., abnormal stress tests, high-risk profiles). Among these, 133 patients were diagnosed with a congenital coronary artery anomaly, representing 1.6% of all angiograms, marginally higher than the ~1.3% rate reported in prior series. The mean age of patients with CAAs was 52 years (range: 18-75 years), with 93 (70%) being male. In most cases (n=123, 93%), the angiography was performed for symptomatic coronary artery disease (CAD) or positive stress findings, leading to incidental discovery of the anomaly. Chest pain was the most frequent presenting symptom (n=80, 60%), followed by dyspnea (n=30, 22.5%) and syncope (n=10,7.5%), while 13 (10%) were asymptomatic (Table 1).

**Table 1:** Summary of clinical presentations

Presentation	Number of patients	Percentage
Chest pain	80	60%
Dyspnea	30	22.50%
Syncope	10	7.50%
Asymptomatic	13	10%

Types of coronary anomalies

Table 2 presents the distribution of the types of CAAs. The most frequent involved the origin of RCA from the left coronary sinus (n=50, 37.6%). The second most common was an anomalous LCx from the right sinus, affecting 40 (30.1%) patients. Together, these two variants (which are generally benign) accounted for ~68% of anomalies. Five patients (3.7%) had an anomalous RCA from the left sinus with an interarterial course between the great arteries, consistent with an “ARCA.” Three patients (2.25%) were identified with a single coronary artery originating from the right sinus, supplying the entire myocardium. In one of these cases, a high-risk intramural course was detected, warranting surgical intervention. Coronary artery fistulas were observed in six (4.5%) patients, and one patient had a high take-off of the RCA. One case of ALCAPA was identified. Certain uncommon anomalies, such as 'inverted coronary arteries,' were categorized observationally, defined specifically in our analysis as coronary arteries originating from opposite coronary sinuses and supplying myocardial territories in a reversed anatomical distribution, without a recognized standardized classification.

**Table 2:** Types and prevalence of coronary artery anomalies ALCAPA: anomalous left coronary artery from the pulmonary artery; RCA: right coronary artery

Anomaly type	Number of patients	Percentage
Anomalous RCA from the left coronary sinus	50	37.60%
Anomalous origin of the circumflex from the right sinus of Valsalva	40	30.10%
Anomalous left main from right sinus of Valsalva	20	15%
Origin of the circumflex coronary artery from the right coronary artery	6	4.50%
Single coronary artery	3	2.25%
Coronary artery fistula	6	4.50%
High take-off RCA	4	3%
ALCAPA	2	1.50%
Inverted coronary arteries	2	1.50%

Notably, benign anomalies dominated the cohort, particularly anomalous RCA originating from the left sinus (n=50, 37.6%) and anomalous LCx arising from the right sinus (n=40, 30.1%), collectively comprising approximately 68% of anomalies. Malignant anomalies represented a smaller proportion, highlighting their comparative rarity.

Imaging modalities

All anomalous coronary arteries in this study were identified and characterized by angiographic imaging. Conventional invasive CAG was the diagnostic modality in the majority of cases. In 106 out of 133 patients (79.6%), the anomaly was initially detected during invasive CAG (often performed due to the patient’s presentation with chest pain or suspected CAD). A smaller subset of patients underwent non-invasive imaging: specifically, 27 (20.4%) patients were diagnosed using CCTA. These 27 patients had their anomalies identified initially and independently through noninvasive imaging, without preceding invasive CAG. Cardiac MRI was not utilized due to limited institutional availability and institutional imaging protocols that preferentially employed invasive angiography and CCTA for comprehensive anatomical delineation. 

Disease status of the anomalous vessel

Assessment of CAD in the anomalous vessel was performed and documented in all patients. In the majority of cases (n=88, 66.1%), the anomalous coronary artery was non-stenotic and free from disease, suggesting that the anomaly itself was not contributing to significant ischemia or obstructive disease. However, among the remaining patients, various degrees of disease within the anomalous vessel were documented. A total of 45 (33.38) patients had significant stenosis of greater than 60%. Eight (6%) patients had total occlusion of the anomalous vessel. Ten patients (7.5%) had severe triple-vessel disease. Among the 45 patients (33.8%) who had significant coronary artery stenosis (>60%), 10 (7.5% of the total cohort) had severe triple vessel disease, representing a subset within the 45 patients with significant stenosis. In all 10 patients diagnosed with triple vessel disease, significant atherosclerotic stenosis involved both anomalous and anatomically normal coronary vessels (Table 3).

**Table 3:** Disease status of the anomalous vessel

Status of anomalous vessel	Number of patients	Percentage
No significant disease	88	66.1
>60% stenosis	45	33.3
Total occlusion	8	6
Severe triple-vessel disease	10	7.5

Intervention strategies

While management approaches for patients with anomalous coronaries varied depending on symptoms and coexistent CAD, most were managed conservatively. Medical management alone was the chosen strategy in the majority of patients; 91 (68.4%) patients received optimal medical therapy with no invasive intervention directed at the anomaly. In these cases, the anomalous vessel either was not causing ischemia or did not require correction, and any mild concomitant CAD was managed with medications. One patient in this medically managed group underwent a concomitant surgical aortic valve replacement (AVR) for severe aortic stenosis, unrelated to the coronary anomaly. The single patient who underwent concomitant surgical AVR did so due to severe aortic stenosis, unrelated directly to the coronary anomaly. The anomaly was incidentally discovered, required no intervention, and thus did not influence the AVR surgical approach.

PCI was performed in 24 (18%) patients. In most of these cases, PCI (typically stent placement) was done to treat a significant atherosclerotic stenosis in a coronary artery rather than to “fix” the anomalous origin itself. A total of 18 patients (13.5%) underwent surgical interventions: 12 patients (9%) underwent coronary artery bypass grafting (CABG) primarily for extensive atherosclerotic disease without direct anomaly correction, while a separate group of six patients (4.5%) required surgical procedures specifically aimed at correcting the anomalous coronary artery (e.g., unroofing, reimplantation). There was no overlap between these two surgical groups. Three of these patients had an interatrial course passing between the aorta and the pulmonary artery, and two of these patients had an intramural course. One patient was diagnosed with ALCAPA, a high-risk anomaly. Surgical correction was performed through reimplantation of the left main coronary artery into the aorta. A summary of management strategies is provided in Table 4.

**Table 4:** Summary of management strategies for patients with anomalous coronary arteries CABG: coronary artery bypass grafting; PCI: percutaneous coronary intervention

Management approach	Number of patients (%)
Medical therapy only (conservative management)	91 (68.4%)
PCI – stenting	24 (18%)
CABG	12 (9%)
Surgical correction of the anomaly (reimplantation)	6 (4.5%)

Immediate procedural outcomes were favorable across management groups: medically managed patients remained stable; PCI was successful without major adverse cardiac events; CABG procedures were completed successfully without immediate postoperative complications; and surgical corrections of anomalies were successful with minor transient complications in one patient.

## Discussion

Key findings

Over a period of 10 years, this comprehensive, retrospective, single-center analysis evaluated a total of 8,000 adult patients who underwent coronary angiography for various clinical indications, predominantly related to suspected or established CAD. In this cohort, 133 patients were diagnosed with congenital CAAs, establishing a prevalence of 1.6%, consistent with previous literature reporting a prevalence of approximately 1.3%-1.6% [1,2]. These anomalies were predominantly incidental findings uncovered during investigations prompted by ischemic symptoms or abnormal noninvasive tests, reflecting their typically silent clinical course [1,2,8]. The two most frequently encountered anatomical variants were an anomalous RCA originating from the left sinus of Valsalva (n=50, 37.6%), and an anomalous LCx arising from the right sinus (n=40, 30.1%). Together, these two variants comprised nearly 68% of all anomalies, reinforcing their status as the most common subtypes encountered in adult patients undergoing coronary evaluations [1,2,12].

The predominant clinical presentation was chest pain (n=80, 60%), followed by exertional dyspnea (n=30, 22.5%) and syncope (n=10, 7.5%). Notably, about 10% of patients were asymptomatic at diagnosis. These findings are consistent with prior studies demonstrating that a significant proportion of CAAs are asymptomatic or cause nonspecific symptoms [5,7]. In our series, invasive coronary angiography was the primary diagnostic method (79.6%), while CCTA identified anomalies in 20.4% of cases. This highlights the growing importance of CCTA as a sensitive and noninvasive modality for the detection and characterization of coronary anomalies [10,11,12]. Assessment of atherosclerotic disease within anomalous vessels revealed that 88 (66.1%) were free of significant stenosis, while significant stenosis (>60%) was present in 44 (33.3%), with complete vessel occlusion in eight (6%). These findings underscore that while CAAs are congenital, they do not inherently protect against or predispose patients to atherosclerotic CAD [12].

The management was predominantly conservative, with optimal medical therapy employed in 91 (68.4%) patients. PCI was performed in 24 (18%), and CABG in 12 (9%). Direct surgical correction of the coronary anomaly was reserved for six (4.5%) patients, primarily those with high-risk features such as an interarterial or intramural course, or severe ischemia-consistent with contemporary international management guidelines [13,14]. Surgical decisions for anomalies with interarterial/intramural courses or ALCAPA were predominantly influenced by symptomatic clinical presentation (exertional chest pain, syncope, ischemia), younger age, and definitive high-risk anatomical imaging features.

Comparison with prior studies

Our observed prevalence of 1.6% aligns closely with large-scale angiographic and CT-based studies. Yamanaka and Hobbs initially reported a prevalence of approximately 1.3% among 126,595 angiograms [1]. Subsequent CT angiographic studies, reflecting enhanced imaging techniques, report slightly higher detection rates (~1.3%-2.0%), suggesting improved detection and characterization with newer modalities [10,12]. The predominance of RCA from the left sinus and LCx from the right sinus in our study is also consistent with previously published data [12].

Symptomatology in our patient population aligns well with published literature, which indicates that CAAs often remain asymptomatic until adulthood and may present with nonspecific symptoms or are discovered incidentally during evaluation for unrelated cardiac conditions [5,7,12]. However, anomalies such as AAOCA with interarterial courses have well-established associations with serious adverse outcomes, including SCD, particularly in young athletes [5,6]. The absence of sudden deaths in our older patient cohort is likely attributable to an older age profile and the increased prevalence of comorbid atherosclerotic disease, potentially mitigating the abrupt presentations observed in younger individuals [15,16].

Diagnostic practices in our study reflect global trends toward multimodality imaging. Although invasive coronary angiography remains the standard diagnostic tool in symptomatic CAD evaluations, the increasing adoption of CCTA is supported by multiple studies highlighting its superior capability in anatomical delineation of CAAs, including proximal vessel course and risk-enhancing anatomical features such as interarterial or intramural trajectories [10,11,12].

From a therapeutic standpoint, our predominantly conservative management strategy aligns with international guidelines recommending medical management for asymptomatic or minimally symptomatic anomalies without high-risk anatomical features [13,15]. Surgical interventions were selectively employed for high-risk CAAs, in accordance with current recommendations, which advocate surgical correction for symptomatic anomalies or those with documented ischemia or high-risk anatomical configurations.

Clinical implications

This study emphasizes the clinical importance of maintaining a high index of suspicion for CAAs during coronary imaging, particularly in younger patients presenting with exertional syncope, unexplained ischemic symptoms in the absence of significant atherosclerosis, unexplained exertional arrhythmias, or atypical angiographic findings such as difficulty locating coronary ostia or unusual coronary artery courses. Accurate diagnosis is critical not only for managing potential ischemia but also for avoiding procedural complications during angiography [8,17]. The high prevalence of benign variants reinforces that many CAAs do not warrant surgical intervention, emphasizing the importance of individualized risk stratification guided by multimodality imaging [13,14,16].

Academically, our data contributes valuable epidemiological insights from a South Asian population, a group historically underrepresented in global CAA research. Notably, this study's demographic profile-characterized by a relatively younger mean age at diagnosis and frequent coexistence of significant atherosclerotic disease-highlights potential differences from Western cohorts, which may reflect distinct genetic, environmental, or lifestyle-related risk factors. These region-specific findings provide important context for international registries and underline the need for further comparative studies to better define ethnic and geographical variations in CAA prevalence, presentation, and outcomes. [16].

Surgical decision-making and anomaly risk stratification followed primarily adult cardiology guidelines, specifically the 2018 ACC/AHA Guidelines for Adults with Congenital Heart Disease and the 2020 ESC Guidelines for Adult Congenital Heart Disease. Pediatric or sports cardiology guidelines were not explicitly utilized in decision-making for this adult cohort. Clinical follow-up was available through detailed review of electronic medical records, capturing procedural success, postoperative recovery, and early symptom resolution. However, due to the retrospective study design, structured long-term prospective follow-up was not systematically conducted, limiting the assessment of long-term clinical outcomes

Role of imaging

Advanced imaging, particularly CCTA, has become integral in diagnosing and risk-stratifying CAAs. Nearly one-fifth of cases in our study were diagnosed exclusively by CCTA, underscoring its value in modern cardiology practice. CCTA provides superior three-dimensional anatomical details, enabling precise characterization of anomalous origins, courses, and anatomical relationships critical for clinical decision-making [12].

Integration of functional imaging modalities, such as stress perfusion imaging and fractional flow reserve CT (FFR-CT), further refines clinical decision-making by distinguishing benign anomalies from those potentially ischemic or arrhythmogenic [15,16]. Cardiac MRI, although highly beneficial in differentiating ischemia from myocardial scarring and assessing viability via late gadolinium enhancement and stress perfusion imaging, was not employed in this cohort primarily due to limited institutional availability and established imaging protocols favoring invasive angiography and CCTA. The absence of CMR may represent a limitation as it could potentially refine the identification of ischemic myocardium, scarring, and viability assessment in anomalous coronary arteries, particularly in patients with ambiguous clinical or anatomical findings.

Limitations

Our study has several inherent limitations stemming from its retrospective, single-center design, which may introduce biases in both data collection and interpretation. Firstly, the variability in diagnostic techniques employed over the study period may have led to inconsistencies in anomaly detection and characterization. Secondly, the lack of routine cardiac MRI and functional assessments, such as FFR or stress testing, in our clinical protocol introduces a notable risk of underdiagnosing or misclassifying high-risk coronary anomalies. These diagnostic tools offer valuable functional and anatomical insights that invasive angiography alone may not adequately capture, especially in the context of asymptomatic individuals. Additionally, our primary reliance on invasive coronary angiography rather than routine utilization of CCTA may further limit the accurate identification and comprehensive three-dimensional delineation of complex coronary anomalies. While invasive angiography provides robust information, its capability to define intricate anatomical relationships is inherently inferior to the detailed three-dimensional reconstructions achievable with CCTA. Lastly, therapeutic decisions in our study were individualized rather than standardized, reflecting practitioner preferences and evolving institutional practices. This variability may reduce the external validity and generalizability of our findings, particularly regarding the prevalence estimates and clinical outcomes reported. Consequently, our reported prevalence figures may differ significantly from the true incidence rates within the broader, particularly asymptomatic, population [17,18,19].

Future directions

Future studies should focus on prospective, multicenter registries to capture diverse populations, facilitating standardized imaging protocols and long-term outcomes assessment. Emerging imaging technologies, including artificial intelligence-based analyses, computational flow modeling, and advanced physiological imaging (such as FFR-CT and 4D flow MRI), hold promise for improving diagnostic accuracy and management precision [20]. Genetic and embryological research could provide insights into congenital pathophysiology, potentially enhancing early identification and management strategies. Development of risk-scoring systems based on anatomical and clinical parameters could further aid individualized decision-making regarding observation, medical management, or intervention [20].

Interdisciplinary collaboration among cardiologists, cardiac imaging specialists, and cardiothoracic surgeons profoundly influenced patient management in this study. A clear example was a patient with ALCAPA, where joint input led to early surgical reimplantation and excellent postoperative outcomes. Such collaborative decision-making aligns with ACC/AHA and ESC guidelines, emphasizing team-based management strategies to optimize patient outcomes.

## Conclusions

Although uncommon, CAAs are clinically significant due to their potential to cause myocardial ischemia, life-threatening arrhythmias, and sudden cardiac death, especially in high-risk variants such as interarterial or intramural courses and ALCAPA. Our study demonstrates a prevalence consistent with contemporary reports and underscores the crucial role of advanced imaging modalities, particularly CCTA, in precise anomaly detection and risk stratification. While prognosis is typically favorable in benign anatomical variants (e.g., anomalous circumflex artery with a benign retroaortic course or anomalous RCA without interarterial/intramural trajectory), high-risk anomalies require vigilant monitoring and often proactive intervention due to elevated risks of adverse cardiac outcomes. Our findings contribute valuable epidemiological data from a South Asian population (a group historically underrepresented in global studies), highlighting unique demographic and clinical features that can inform international registries and aid in refining clinical guidelines. However, the retrospective and single-center design, along with the absence of systematic long-term follow-up, are important limitations affecting generalizability. Future guidelines should incorporate precise anatomical and functional imaging criteria for improved risk stratification, clearer evidence-based thresholds for surgical intervention, and tailored surveillance protocols. Ultimately, standardized scoring systems integrating clinical, anatomical, functional, and genetic parameters hold the promise to significantly enhance personalized clinical decision-making and patient outcomes in the management of CAAs.
